# Fluoride varnish, ozone and octenidine reduce the incidence of white spot lesions and caries during orthodontic treatment: randomized controlled trial

**DOI:** 10.1038/s41598-022-18107-w

**Published:** 2022-08-17

**Authors:** Katarzyna Grocholewicz, Paulina Mikłasz, Alicja Zawiślak, Ewa Sobolewska, Joanna Janiszewska-Olszowska

**Affiliations:** 1 Department of Interdisciplinary Dentistry, Pomeranian Medical University, Al. Powstańców Wlkp. 72, 70-111 Szczecin, Poland; 2 Institute of Mother and Child, Warsaw, Poland; 3 Department of Dental Prosthetics, Pomeranian Medical University, Al. Powstańców Wlkp. 72, 70-111 Szczecin, Poland

**Keywords:** Oral diseases, Preventive dentistry

## Abstract

This randomized, parallel, controlled trial assessed the effect of fluoride varnish, ozone and octenidine on white spot lesions (WSLs) and caries during orthodontic treatment. Patients were enrolled between 1st September 2017 and 31st August 2020 at initiation of orthodontic treatment in Department of Interdisciplinary Dentistry Pomeranian Medical University in Szczecin, Poland. All participants were randomly assigned to four study and one control groups using number random generator. However, investigators were not blinded due to the nature of the study. Groups I, II, III, IV had professional cleaning and varnishing (5% NaF) every 4 weeks. Groups II and IV had in-office ozone therapy before varnishing, groups III and IV received domestic octenidine mouthrinse. Group K had no professional hygienic or prophylactic procedures. WSLs were assessed at T0 and then every 4 weeks (T1–T4) and caries—at T0 and T4. The specific objective was to assess the influence of fluoride varnish, ozone and octenidine on the incidence of white spot lesions and caries during orthodontic treatment. The primary outcome of this report was the highest number of WSLs in group K and the lowest percentage of patients with WSLs in group IV. Each group comprised 30 randomized participants; they were all analyzed. No WSLs were found at T0, but they were stated in all groups at T4. The numbers of patients with WSLs significantly increased between T0-T4 in groups I and K. Group IV had the lowest percentage of patients with WSLs in T1-T4. WSLs in group IV were found no earlier than at T2. Group K had the highest percentage of WSLs at T4: 26%. At T0 all the groups had DMFs above 0 with a significant increase at T4. No side effects of the introduced prophylaxis were observed in any group. Caries is an important problem of fixed orthodontic treatment. Even an extremely intensive prophylaxis could not completely prevent WSLs and caries. Simultaneous application of fluoride varnish, ozone gas exposure and octenidine appears to have a beneficial effect in limiting the development of WSLs.

Trial registration: NCT04992481.

## Introduction

Orthodontic treatment has become very popular in recent years. Appearance has become extremely important in the modern society. Thus, patients seek orthodontic treatment, mainly to improve the esthetics of their smiles. Another reason to have orthodontic treatment is striving for a better, more comfortable occlusion. However, complications of orthodontic therapy may occur.

Bonding an orthodontic appliance enhances bacterial adhesion due to new retention sites with reduced salivary flow. The most retentive sites are: area between the bracket and gingival margin and proximal surfaces. The resulting pH reduction promotes demineralization^1^. Enamel demineralization and gingival inflammation belong to the most prevalent detrimental effects resulting from increased accumulation of the biofilm and difficulty to clean these surfaces.

Following treatment initiation, the microbial environment of the oral cavity changes—the number of *Lactobacillus* and *Streptococcus* *mutans* in biofilm increases^2^.

White spot lesions (WSLs) result from demineralization and are the first sings of enamel carious lesions that can be detected with the naked eye^3^. WSLs may arise as early as four^4^ or even 2 weeks^5^ following fixed appliance bonding^1,6^.

The most often affected teeth are lateral incisors^7^, followed by canines, premolars and central incisors^8,9^. There is no side predilection, or difference for upper and lower dental arches^7,8,10^. At least one new initial carious lesion is found in 72.9% patients^11^. Cavities develop during the first 12 months of orthodontic treatment in 61% patients^12^. A rapid increase of the incidence of WSLs is observed during the first 6 months of treatment—initial caries is found in 38% patients, after 12 months WSLs are found in 46% patients.

Caries prevention in patients under orthodontic treatment is based on hygienic regimen. Biofilm accumulation can be reduced by proper brushing technique, use of fluoride, as well as bactericidal or remineralizing chemicals: chlorhexidine, octenidine, ozone or casein phosphopeptides-amorphous calcium phosphate (CPP-ACP)^13–21^.

The alterations of enamel structure within WSLs are reversible only partially. Even 12 years after debonding the number of WSLs does not return to that from before orthodontic treatment^8^. The treatment methods comprise: remineralization (with fluoride or hydroxyapatite), resin infiltration, microabrasion or restorative treatment, depending on the severity of the lesion^13,22–26^.

WSLs constitute a health and an esthetic problem—introducing preventive measures and early detection of WSLs is essential. Patients prepared for orthodontic treatment should have a high hygienic regimen.

Professional fluoride prophylaxis, using a varnish or gel should be considered in patients with increased caries risk^27^. In-office application of fluoride varnish allows for a control of application and is independent from patient’s domestic cooperation^28,29^. A meta-analysis of 36 RCTs showed NaF varnish is most effective in prevention of orthodontically induced WSLs^22^. Application of fluoride varnish on every orthodontic appointment reduces enamel demineralization by 44% comparing to placebo^13^.

Ozone is a strong oxidant, destructive to various pathogens^30,31^; exerts bactericidal, antiviral and fungicidal activity as well as stimulates tissue metabolism by increasing oxidation^25,32^. It enables remineralization and reduces inflammation, by stimulating interleukin and interferon production, breaking protein chains and oxidating aminoacids. Moreover, ozone removes residual biomolecules, mostly acids, that help to perpetuate the acidogenic niche. Ozone has been proved to exert many beneficial effects in the treatment of oral diseases^33,34^. It exerts an antimicrobial effect in carious lesions following partial removal of caries^35^, similar to 2% chlorhexidine solution^36^. Antimicrobial effect has even been shown for primary root carious lesions^37,38^. The treatment of deep carious lesions with almost exposed vital pulp and pain (excluding at night) was associated with less pain during the following 24 h and a lower percentage of root canal treatment, if ozone was applied following partial caries removal, then if traditional complete caries removal was proceeded^39^. Tooth bleaching was more effective and caused less hypersensitivity, if ozone was applied at the end of the bleaching session^40^.

Octenidine dihydrochloride influences the biofilm by exerting antimicrobial effect on: *S*. *mutans*, *S*. *sanguis*, *A. viscosus*, *A. naeslundii*^41^.

The aim of the present study was to assess the influence of fluoride varnish, ozone and octenidine on the incidence of white spot lesions and caries during orthodontic treatment.

## Material and methods

A single-center, parallel, RCT was conducted in Department of Interdisciplinary Dentistry Pomeranian Medical University in Szczecin, Poland. Initially, 167 consecutive adult patients (16–50 years) undergoing orthodontic treatment were included in the study. All participants began orthodontic treatment with fixed appliances bonded to both dental arches were eligible and consecutively invited. Seventeen patients declined to participate in the research. Informed consent was obtained from 150 patients (111 women and 39 men) (Table 2). In patients under 18 years of age, informed consent for study participation was obtained both from the patient and from a parent and/or legal guardian (according to national law regulations). No criteria for the planned study were changed after study initiation. Ethical approval for this randomized clinical trial has been granted by Bioethic Committee of Pomeranian Medical University in Szczecin, with a reference number: KB-0012/124/12. The study has been registered at ClinicalTrials.gov, Identifier: NCT04992481, registration date: 05.08.2021 and all methods were performed in accordance with the relevant guidelines and regulations.

The following exclusion and inclusion criteria were applied:

Inclusion criteria:

- age 16–50 years
- permanent dentition
- generally healthy patients
- initiation of fixed orthodontic treatment with brackets bonded to both dental arches

Exclusion criteria:

- developmental dental abnormalities
- prosthetics restorations
- general diseases
- medical problems that might affect salivary flow

Sample size calculation for repeated-measures ANOVA was performed by using the R function wp.rmanova, WebPower package^42^. The power of correlation was established at the level of 0.75. Given the power, effect size, the minimum sample size can be calculated as shown in Table 1.

**Table 1:** Input and output for sample size planning for repeated-measures ANOVA. ()

f	ng	nm	nscor	Alpha	Power	n
0.25	5	2	0.7	0.05	0.75	144.4

*n* sample size, *ng* number of groups, *nm* number of measurements, *f* effect size (prior medium value = 0.25), *nscor* sphericity correction coefficient (0.7 for non-sphericity data), *alpha* significance level, *type* type of effect analysis (1 for within type), *power* statistical power.

The patients have been randomly divided (using number random generator, provided by the second author) into five groups (n = 30), including four study groups (I, II, III, IV) and one control group (K). Distribution of the study and control groups according to sex has been presented in Table 2.

**Table 2:** Distribution of the study and control groups according to sex.

Group	Men		Women	
	n	%	n	%
I	10	33.33	20	66.67
II	7	23.33	23	76.67
III	8	26.67	22	73.33
IV	7	23.33	23	76.67
K	7	23.33	23	76.67
Total	39	26	111	74

Detailed initial oral hygiene instruction recommendations were provided to all patients. The recommendations comprised: toothbrushing every surface after every meal (4 min) with a fluoridated toothpaste (1450 ppm) using the roll and Bass methods with a soft toothbrush recommended for fixed appliance, interdental toothbrushes, single-tufted brushes and dental floss recommended for fixed appliance. An instruction leaflet has been delivered to every subject, as well. A reinstruction was made during every control appointment referring to the results of the oral hygiene assessment.

**Figure 1:**
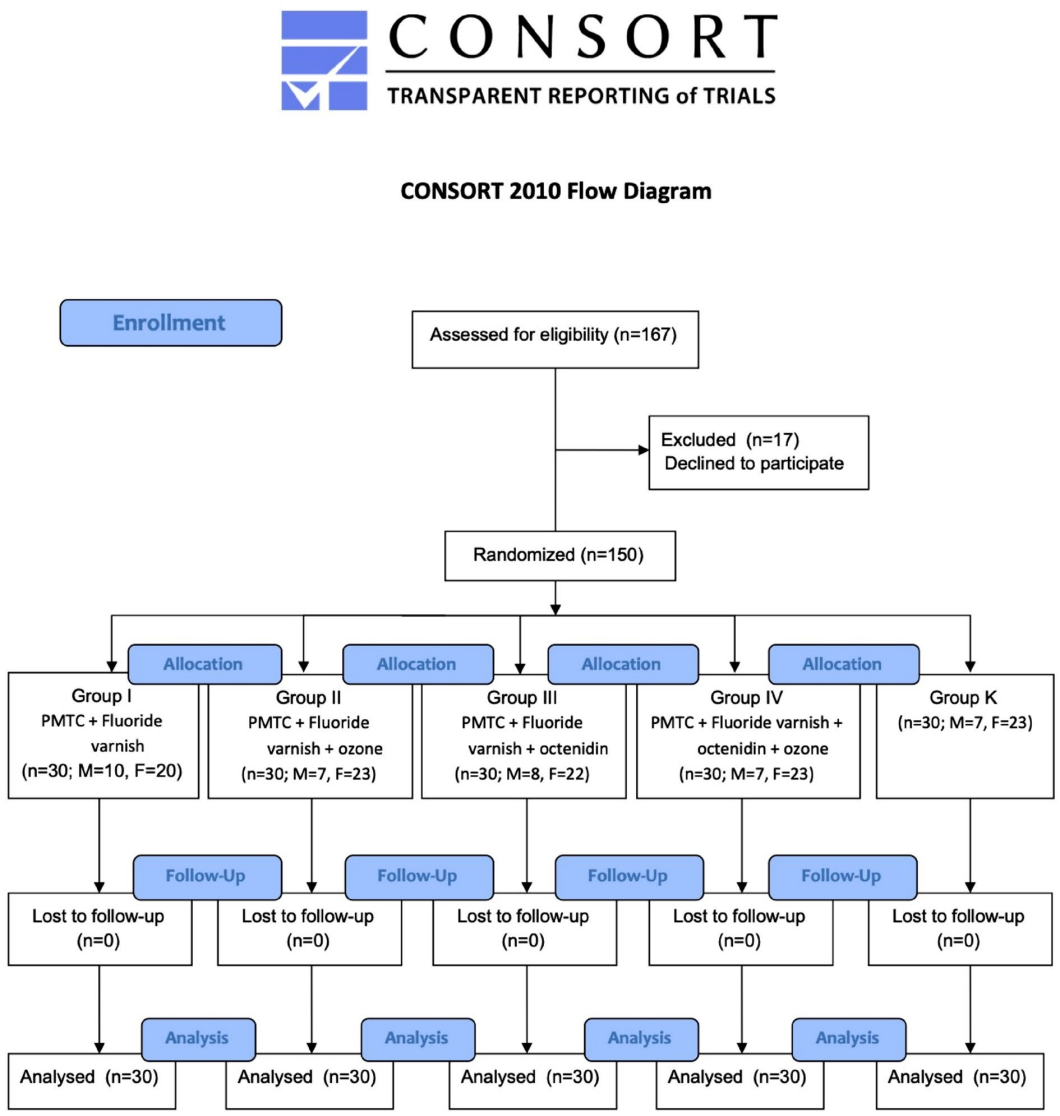
Consort flow diagram.

The distribution of prophylaxis procedures (performed every 4 weeks) in the study and control groups has been presented in Consort flow diagram (Fig. 1).

In patients from the study groups I, II, III and IV professional mechanical tooth cleaning (PMTC) including air-polishing was proceeded on every appointment using a glycine-based prophylaxis powder (Air-Flow Soft, EMS, Switzerland).

In patients from groups I, II, III and IV a fluoride varnish of 5% NaF (Profluorid Varnish, Voco, Germany) was applied with a microbrush. Every patient was instructed to stop from drinking and eating for the next two hours (as recommended by the manufacturer).

Patients from groups II and IV were additionally subjected to in-office ozone therapy using OzonyTron-OZ (Mymed, Germany). Ozone was applied on a silicone double-jaw tray of a proper size (allowing even ozone distribution in the oral cavity) following professional mechanical tooth cleaning. A Full Mouth Disinfection program lasting 5 min was used. According to the manufacturer, ozone concentration in the oral cavity during full mouth disinfection programme is 0.97 ppm. For patient’s safety the silicone tray of a proper size was adapted to tightly adhere to the dental arch.

Patients from groups III and IV received a domestic mouthrinse (Octenidol MD, Schülke, Germany) containing water, PEG-40, hydrogenated castor oil, glycerin, flavor, sodium gluconate, sucralose, octenidine hydrochloride, citric acid and BHT and were recommended to use 15 ml of the liquid for 30 s after evening toothbrushing.

Patients assigned to control group had no professional hygienic or prophylactic procedures performed, except for initial oral hygiene instruction and written dietetic recommendations.

The period of observation was 12 months, with prophylactic visits every 4 weeks and examination every 12 weeks. The assessment of white spot lesions was proceeded on the labial surfaces of upper and lower teeth, with patient on the dental chair in proper dental lighting, after cleaning and drying the assessed surfaces. The clinical examination was performed by the same experienced investigator*.* WSL index according to Gorelick et al.^43^ was used as follows:

- 0—no visible WSL or surface disruption (no demineralization).
- 1—visible WSL, covering less than 1/3 of surface, without surface disruption (mild demineralization).
- 2—visible WSL, covering more than 1/3 of the surface, with a roughened surface, but not requiring restoration (moderate demineralization).
- 3—visible cavitation, requiring restoration (severe demineralization).

Every dental surface was subjected to visual-tactile examination performed on a dental chair with proper dental lighting, using dental probe and a mirror. Caries was diagnosed on the level of cavitation, the presence of fillings was noted as well. The data obtained in examinations (t0–t4) were recorded on a specially developed patient examination chart. All examinations and prophylactic procedures were performed by the same researcher (PM).

### Statistical analysis

All continuous variables were verified for distribution normality using Kołmogorov–Smirnov test, Shapiro–Wilk test or qq plots. Statistical significance of differences between two groups were analyzed using Student *t* test and Mann–Whitney test. Multiple group comparisons were made using analysis of variance (ANOVA), Tukey HSD test or Kruskal–Wallis test^44–46^.

Discontinuous variables were described by their number and frequency of occurrence. Statistical correlations between discontinuous variables were analyzed using χ^2^ Pearson’s test or exact Fisher’s test.

For the analysis of correlations between discontinuous variables: ordinal and nominal (coded variables: 0/1) and continuous variables rank Spearman correlation coefficient was used. The correlations were described using the r correlation coefficient and the level of significance p.

Statistical analysis with data visualization has been programmed in the interpreted programming language “R”^47^ with open-source software for data science, scientific research, and technical communication “R studio” version 1.4.1106^48^. The level p = 0.051–0.099 has been set as a borderline trend of statistical significance. Computer software STATA 11 (2009) of license No 30110532736 has been used for all analyses.

## Results

A total sample size of 144.4 was needed to obtain a power of 0.75. Since the sample size has to be an integer and also divisible by 5, the minimum sample size required was 145 or 29 subjects for each group. No losses or exclusions after randomization occurred.

The incidence of WSLs in the study and control groups in subsequent examinations are shown in Table 3.

**Table 3:** Number of patients with WSLs in the study and control groups in subsequent examinations.

Group	Examination	n (%)	T0 versus T4
			P
I	T0	0 (0.00)	0.01777
	T1	2 (6.67)	
	T2	5 (16.67)	
	T3	5 (16.67)	
	T4	5 (16.67)	
II	T0	0 (0.00)	0.05590 (NS)
	T1	1 (3.33)	
	T2	3 (10.00)	
	T3	3 (10.00)	
	T4	3 (10.00)	
III	T0	0 (0.00)	0.06478 (NS)
	T1	1 (3.33)	
	T2	3 (10.00)	
	T3	3 (10.00)	
	T4	3 (10.00)	
IV	T0	0 (0.00)	0.05414 (NS)
	T1	0 (0.00)	
	T2	1 (3.33)	
	T3	2 (6.67)	
	T4	2 (6.67)	
K	T0	0 (0.00)	0.00040
	T1	2 (6.67)	
	T2	4 (13.33)	
	T3	7 (23.33)	
	T4	8 (26.67)	

At T0 examination, no patients with WSLs were found in any of the groups examined. After a year of orthodontic treatment, WSLs were stated in all groups observed. The lowest percentage of patients with WSLs was seen in group IV in all examinations. Moreover, WSLs in patients from group IV were found no earlier than T2 examination—6 months after initiation of the study. The highest increase of the number of WSLs with time was noted in group K. After a year of follow-up, WSLs were found in 26% of the examined patients. Statistically significant differences were found concerning the numbers of patients with and without WSLs between T0 and T4 in groups I (p = 0.01777) and K (p = 0.0004).

**Figure 2:**
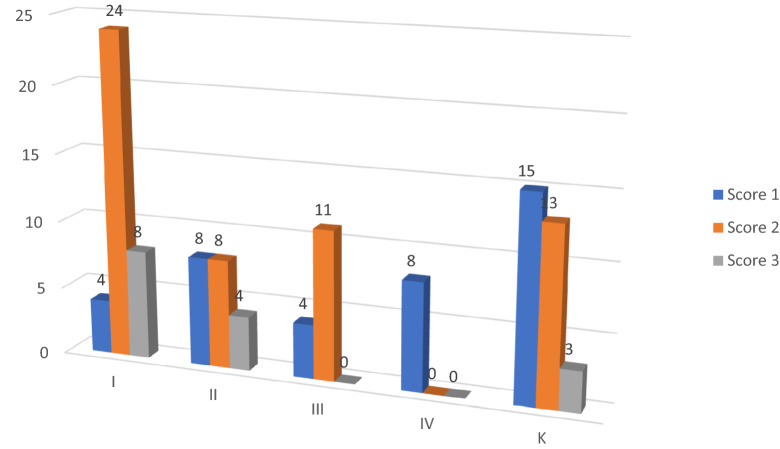
Distribution of the severity and number of WSLs in the study and control groups at the final examination (T4).

Distribution of the severity of WSLs in the final examination is presented in Fig. 2.

The lowest number of WSLs was found in group IV (8), the highest—in group I (36). According to WSL index the highest number of white spite lesions was noted in the score 2 (46). More severe lesions were noted in groups I and K. In group IV there were observed the lowest number of WSLs and all were scored 1.

Distribution of DMF values in T0 and T4 examinations in the groups examined has been presented in Table 4.

**Table 4:** Basic descriptive statistics of T0, T4 DMFs values (Time) by a grouping variable (Group).

Group	Examination	Mean	SD	Median	Min	Max	Q1	Q3
I	T0	0.15	0.13	0.10	0	0.40	0.04	0.23
II		0.12	0.08	0.10	0	0.40	0.07	0.18
III		0.11	0.08	0.10	0	0.32	0.05	0.12
IV		0.09	0.07	0.06	0.02	0.26	0.04	0.13
K		0.15	0.12	0.13	0	0.40	0.06	0.20
I	T4	0.17	0.14	0.12	0.01	0.43	0.04	0.23
II		0.16	0.11	0.12	0.02	0.5	0.09	0.21
III		0.13	0.09	0.11	0	0.34	0.06	0.20
IV		0.10	0.07	0.7	0.03	0.26	0.04	0.14
K		0.19	0.12	0.17	0.02	0.50	0.08	0.24

Statistically significant differences in DMF values within the study groups between T0 and T4 are presented in Table 5.

**Table 5:** Statistical significance for *T0, T4* pairwise comparisons within each group by using sign test (*pairwise_sign_test*(), {*rstatix*} package).

Groups	n1	n2	df	p	p.adj
I	30	30	16	< 0.001	< 0.001
II			16	< 0.001	< 0.001
III			15	< 0.001	< 0.001
IV			8	0.008	0.008
K			27	< 0.001	< 0.001

*n1*, *n2* sample counts for T0, T4 respectively, *df* the total number of differences (degrees of freedom), *p* p-value, *p.adj* -value adjusted by “holm” method.

DMF values above 0 were stated in all the groups examined at T0. During the follow-up period DMF values significantly increased in all the study groups. No cavities have been found on the labial or buccal surfaces in any in the patients examined.

## Discussion

Caries and gingival inflammation belong to the most prevalent complications of orthodontic treatment^49^. Orthodontic patients are particularly vulnerable to increased bacterial plaque deposition due to dental abnormalities and the attachments of the orthodontic appliance creating additional retention sites^50,51^.

It should be noticed that score 3 of the severity of WSLs is equivalent to carious decay on the labial or buccal surface. Therefore, the same lesion could theoretically be registered twice: in WSLs score and in DMFs. However, in the present study no cavities have been diagnosed on the labial or buccal surface in any of the patients included. Otherwise, double reporting would occur. It could be discussed if score 3 could be removed from the WSL index since its description (visible cavitation, requiring restoration) does not meet the definition of a white spot lesion (chalky-white area resulting from subsurface demineralization).

The increasing incidence of WSLs in the present study is caused by compromised oral hygiene during fixed orthodontic treatment^52^. Abbate et al.^53^ have found an increase of PI (Plaque Index) within 12 months follow-up on incisors (from initial value of 0.72 to 2.32) and on molars (from 0.92 to 2.52). Other studies reported an increased incidence and severity of early carious lesions caused by orthodontic therapy, with the percentage of patients who developed caries during orthodontic treatment ranging between 2 and 96%^43,54,55^. Carious lesions are usually found on maxillary molars and lateral incisors as well as on mandibular canines and premolars^54^.

In the study by Øgaard et al.^56^ on 40 patients, who developed WSLs during orthodontic treatment, a recovery of mild demineralization lesions occurred in 75% in a 6-years follow-up after cessation of orthodontic therapy, whereas more severe lesions (present in 25% patients) were still visible. Thus, it must be underlined that WSLs may recover, depending on their severity. However, at the moment, there is no method that would allow to completely reverse demineralization at various stages of advancement^19,20,29,57,58^. Therefore, prophylaxis should gain a high priority.

The most popular method of domestic prophylaxis is the use of fluoridated toothpaste. Singh et al.^58^ showed, that the use of 1000 ppm of fluoride toothpaste twice daily was effective in the remineralization of postorthodontic WSLs and that additional use of 5% NaF varnish or CPP-ACP had furthermore no beneficial effect in the remineralization. In a study on premolars extracted for orthodontic reasons in 20 patients, O’Reilly et al.^59^ proved the effectiveness of a toothpaste containing NaF (1100 ppm) and a mouth rinse containing 0.05% NaF and 1.2% APF (acidulated phosphate fluoride—sodium fluoride acidified with phosphoric acid) in stabilizing and inhibiting demineralizing lesions. However, Geiger et al.^15^ reported that only 15% of the patients follow the recommendation to rinse their mouths. On the other side, Øgaard et al.^60^ reported that regular use of toothpaste with fluoride as the only method of caries prevention is ineffective. These findings have been confirmed by the results from control group of the present study.

Thus, where domestic oral hygiene does not prevent demineralization, professional individual prophylactic procedures should be introduced. According to Arends et al.^61^, fluoride varnish stays longer on the tooth surface and is more effective than using a toothpaste with fluoride only. Shen et al.^62^ in their in vitro study, on the effectiveness of fluoride varnish, reported a higher effectiveness of varnishes containing only fluoride compared to varnishes containing fluorine ions and calcium phosphate. Sonesson et al.^28^, in an in vivo study, observed that professional application of fluoride varnish during fixed orthodontic treatment exerted a minor long-term effect on advanced WSLs on surface level. Recent systematic reviews showed that the use of fluoride varnish is one of the most common and effective methods of preventing WSLs^18,19,63^. Thus, the results of previous studies are contradictory. In the present study, patients who had only Profluorid Varnish applied (group I) had a higher number of WSLs than patients subjected to combined prophylactic measures (groups II, III, IV), but lower than the control group.

Early carious lesions may develop within a month from bonding a fixed appliance^43,54^. Various percentages were reported in the literature: Gorelick et al.^43^ found WSLs in 50% patients. Richter et al.^64^ noticed the development WSL in 73% of 350 patients, who had no prophylactic measures introduced. Lovrov et al.^65^ have observed new WSLs in 25% of 53 patients, who had fluoride varnish (Elmex Gelee, GABA, Germany) applied every 6 months and were administered domestic use of Elmex Fluid (GABA, Germany) once a week. Ahmed et al.^66^ in their study on 90 patients found 33% carious lesions after 6 months and 61% after a year of follow-up from the beginning of orthodontic treatment.

In the study by Enaia et al.^67^ WSLs were reported in 61% of 400 patients, who had no prophylactic measures introduced. In the present study WSLs were reported in 26.67% of patients from the control group (with no prophylactic measures introduced). A lower percentage of patients with new WSLs was noted in study groups who had prophylactic measures introduced, e.g. 6.67–16.67%, depending on the group. In the control group WSLs in the grades 2 or 3 were observed in 6.67% of patients, and in grade 1—in 13.3%. In the study by Enaia et al. grade 3 WSLs were found in 1.8%, grade 2 WSLs in 13.3%, grade 1 WSLs in 45.8%. Consistent results concerning a lower percentage of mild versus severe lesions were reported by Sonesson et al.^28^, who compared 75 patients with fixed maxillary appliances, subjected to fluoride varnishing every 6 weeks to 73 patients from an unexposed control group. A year later WSLs were found in 19.6% (score 2) and 3.5% (score 3) patients form study group. In the control group the number of WSLs was 15.8% (score 2) and 6.2% (score 3). Over 70% of patients from both groups had score 1. It can be supposed that small differences between the results may be caused by diverse sizes of the study groups. It should be underlined that according to the study by Øgaard et al. WSLs scored 2 do not recover in a long-term follow-up^56^.

WSLs arise in orthodontic patients despite domestic oral hygiene, with fluoridate toothpaste and fluoridated mouthrinse^68–70^. However, it has been proved that fluoride has an influence on the incidence of WSLs^16,71–73^. These results are consistent with the present study: the use 5% NaF resulted in a reduced incidence of WSLs in all the groups examined comparing to control group.

Kronenberg et al.^74^ in their 26 months split-mouth study applied: Cervitec, Fluor Protector or ozone therapy in different quadrants of the dentition, whereas one quadrant served as control. In the quadrants where Cervitec or Fluor Protector were used, the incidence of WSLs increased by a mean of 0.7% surfaces. In the quadrants where ozone therapy was used, the increase of WSLs incidence was 3.2%. Thus, varnishes with fluoride or chlorhexidine exert a higher anti-carious effectiveness than ozone therapy. Kronenberg et al.^74^ used Cervitec or Fluor-Protector varnish. In the present study combining ozone therapy with 5% NaF varnish in group II and rinse with octenidine in group IV resulted in the lowest percentage of WSLs compared to the other groups.

Very few studies could be found referring to remineralizing effect of ozone therapy. Huth et al.^75^ reported that ozone application has a remineralizing effect on non-cavitated fissure caries. Al-Shamsi confirmed a preventive effect of ozone therapy in orthodontic patients^76^. According to Kronenberg et al.^74^ monotherapy with ozone is less effective than fluoride varnish. The remineralizing effect of combined ozone therapy and nanohydroxyapatite has been reported in a study by Grocholewicz et al.^77^ on 92 patients (age 20–30 years) with initial caries lesions (diagnosed on bite-wings radiographs) on proximal surfaces of posterior teeth (n = 546). In 1-year therapy remineralization was found in 36.5% lesions in ApaCare group, 60% in Ozone group and 69.3% in ApaCare + Ozone group.

No scientific clinical studies reporting effect of octenidine on caries could be found. Dogan et al.^78^ analyzed the effect of octenidine rinse in 18 orthodontic patients, who received: physiologic saline solution, a mouthrinse with octenidine, a mouthrinse with chlorhexidine or a mouthrinse with iodine polyvidone (PVP-I) for 5 days with 2 weeks intervals between the solutions. It was concluded that octenidine exerts a stronger effect in terms of reducing the total number of *Lactobacillus* *S. mutans* comparing to chlorhexidine or PVP-I. Thus, it can be assumed that octenidine, may be considered as a beneficial antiseptic in patients treated with fixed appliances.

In the present study the most beneficial prophylactic effect throughout the whole follow-up period was noted in group IV, subjected to all the prophylactic procedures, including ozone therapy, application of 5% NaF varnish and domestic mouth rinse containing octenidine. The use of combined prophylactic measures allowed to reduce effect of deteriorated oral hygiene. The dental status was better compared to patients who had no prophylactic measures introduced. Possible limitations of the present study may refer to patients’ cooperation in terms of following oral hygiene instructions and the use of domestic mouthrinse.

Moreover, beneficial effects resulting from frequent professional cleaning procedures as well as oral hygiene instructions with demonstration on a model and reinstructions allowing to verify incorrect toothbrushing technique, should be underlined. In the future introducing automatic objective detection and assessment of white spot lesions based on artificial intelligence may bring important progress in diagnosing and monitoring early stages of caries in patients under orthodontic therapy^79^.

## Conclusion

Caries incidence is an important problem of patients undergoing fixed orthodontic treatment.

Even an extremely intensive prophylactic program could not completely prevent WSLs and carious lesions. Simultaneous application of fluoride varnish, ozone gas exposure and octenidine appears to have a beneficial effect in limiting the development of WSLs.

Efforts should be made to prevent new caries lesions during orthodontic treatment.

## Data Availability

Raw data is available from the corresponding author on a reasonable request.

## References

[1] Øgaard B (2008). White spot lesions during orthodontic treatment: Mechanisms and fluoride preventive aspects. Semin. Orthod..

[2] Scheie A, Arneberg P, Krogstad O (1984). Effect of orthodontic treatment on prevalence of *Streptococcus*
*mutans* in plaque and saliva. Scand. J. Dent. Res..

[3] Fejerskov O, Nyvad B, Kidd E, Fejerskov O, Kidd E (2003). Clinical and histological manifestations of dental caries. Dental Caries: The Disease and Its Clinical Management.

[4] Øgaard B, Rolla G, Arends J (1988). Orthodontic appliances and enamel demineralization. Part 1. Lesion development. Am. J. Orthod. Dentofac. Orthop..

[5] Panuszka J, Zarzecka J, Stós W (2006). Higiena jamy ustnej i profilaktyka choroby próchnicowej zębów pacjentów leczonych aparatami stałymi. Porad. Stomatol..

[6] Balenseifen J, Madonia W (1970). Study of dental plaque in orthodontic patients. J. Dent. Res..

[7] Tufekci E, Dixon SJ, Gunsolley JC, Lindauer SJ (2011). Prevalence of white spot lesions during orthodontic treatment with fixed appliances. Angle Orthod..

[8] Shungin D, Olsson A, Persson M (2010). Orthodontic treatment-related white spot lesions: A 14-year prospective quantitative follow-up, including bonding material assessment. Am. J. Ortho Dentofac. Orthop..

[9] Kozak U, Sękowska A, Chałas R (2020). The effect of regime oral-hygiene intervention on the incidence of new white spot lesions in teenagers treated with fixed orthodontic appliances. Int. J. Environ. Res. Public Health.

[10] Chapman J, Roberts W, Eckert GJ, Kula K, González-Cabezas C (2010). Risk factors for incidence and severity of white spot lesions during treatment with fixed orthodontic appliances. Am. J. Orthod. Dentofac. Orthop..

[11] Maxfield B, Hamdan A, Tüfekçi E, Shroff B, Best A, Lindauer S (2012). Development of white spot lesions during orthodontic treatment: Perceptions of patients, parents, orthodontists, and general dentists. Am. J. Orthod. Dentofac. Orthop..

[12] Derks A, Kuijpers-Jagtman A, Frencken J, Van’t Hof M, Katsaros C (2007). Caries preventive measure used in orthodontic practices: An evidence-based decision?. Am. J. Orthod. Dentofac. Orthop..

[13] Stecksen-Blicks C, Renfors G, Oscarson N, Bergstrand F, Twetman S (2007). Caries-preventive effectiveness of a fluoride varnish: A randomized controlled trial in adolescents with fixed orthodontic appliances. Caries Res..

[14] Øgaard B, Larsson E, Henriksson T, Birkhed D, Bishara SE (2001). Effects of combined application of antimicrobial and fluoride varnishes in orthodontic patients. Am. J. Orthod. Dentofac. Orthop..

[15] Geiger A, Gorelick L, Gwinnett A, Benson BJ (1992). Reducing white spot lesions in orthodontic populations with fluoride rinsing. Am. J. Orthod. Dentofac. Orthop..

[16] Demito C, Vivaldi-Rodriguez G, Ramos A, Bownan S (2004). The efficacy of a fluoride varnish in reducing enamel demineralization adjacent to orthodontic brackets: An in vitro study. Orthod. Craniofac. Res..

[17] Msallam F, Grawish M, Hafez A, Abdelnaby Y (2017). Decalcification prevention around orthodontic brackets bonded to bleached enamel using different topical agents. Prog. Orthod..

[18] Sharda S, Gupta A, Goyal A, Gauba K (2021). Remineralization potential and caries preventive efficacy of CPP-ACP/Xylitol/Ozone/Bioactive glass and topical fluoride combined therapy versus fluoride mono-therapy—A systematic review and meta-analysis. Acta Odontol. Scand..

[19] Höchli D, Hersberger-Zurfluh M, Papageorgiou SN, Eliades T (2017). Interventions for orthodontically induced white spot lesions: A systematic review and meta-analysis. Eur. J. Orthod..

[20] Tahmasbi S, Mousavi S, Behroozibakhsh M, Badiee M (2019). Prevention of white spot lesions using three remineralizing agents: An in vitro comparative study. J. Dent. Res. Dent. Clin. Dent. Prospects.

[21] Lale S, Solak H, Hınçal E, Vahdettin L (2020). In vitro comparison of fluoride, magnesium, and calcium phosphate materials on prevention of white spot lesions around orthodontic brackets. Biomed Res. Int..

[22] Hu H, Feng C, Jiang Z, Wang L, Shrestha S, Yan J, Shu Y, Ge L, Lai W, Hua F, Long H (2020). Effectiveness of remineralizing agents in the prevention and reversal of orthodontically induced white spot lesions: A systematic review and network meta-analysis. Clin. Oral Investig..

[23] Prasada K, Penta P, Ramya K (2018). Spectrophotometric evaluation of white spot lesion treatment using novel resin infiltration material (ICON). J. Conserv. Dent..

[24] Lee J, Okoye L, Lima P, Gakunga P, Amaechi B (2020). Investigation of the esthetic outcomes of white spot lesion treatments. Niger. J. Clin. Pract..

[25] Dai Z, Liu M, Ma Y, Cao L, Xu H, Zhang K, Bai Y (2019). Effects of fluoride and calcium phosphate materials on remineralization of mild and severe white spot lesions. Biomed Res. Int..

[26] Paula A, Fernandes A, Coelho A, Marto C, Ferreira M, Caramelo F, do Vale F, Carrilho E (2017). Therapies for white spot lesions—A systematic review. J. Evid. Based Dent. Pract..

[27] Benson P, Parkin N, Dyer F, Millett D, Germain P (2019). Fluorides for preventing early tooth decay (demineralised lesions) during fixed brace treatment. Cochrane Database Syst. Rev..

[28] Sonesson M, Twetman S, Bondemark L (2014). Effectiveness of high-fluoride toothpaste on enamel demineralization during orthodontic treatment—A multicenter randomized controlled trial. Eur. J. Orthod..

[29] Sonesson M, Brechter A, Abdulraheem S, Lindman R, Twetman S (2020). Fluoride varnish for the prevention of white spot lesions during orthodontic treatment with fixed appliances: A randomized controlled trial. Eur. J. Orthod..

[30] Chomicz A, Pietruska M (2007). Zastosowanie ozonu w leczeniu stomatologicznym. Mag. Stomatol..

[31] Iwanek P (2007). Biologiczne podstawy działania ozonu na florę jamy ustnej. Ann. Acad. Med. Stetin..

[32] Grootveld M, Silwood CJ, Lynch E (2006). High resolution 1H NMR investigations of the oxidative consumption of salivary biomolecules by ozone: Relevance to the therapeutic applications of this agent in clinical dentistry. BioFactors.

[33] Zanardi I, Borrelli E, Valacchi G, Travagli V, Bocci V (2016). Ozone: A multifaceted molecule with unexpected therapeutic activity. Curr. Med. Chem..

[34] Smereka, J. & Aluchna, M. Zastosowanie ozonoterapii w stomatologii, *Forum Stomatologii Praktycznej* 3–4 (2016).

[35] Libonati A, Di Taranto V, Mea A, Montemurro E, Gallusi G, Angotti V, Nardi R, Paglia L, Marzo G, Campanella V (2019). Clinical antibacterial effectiveness Healozone Technology after incomplete caries removal. Eur. J. Paediatr. Dent..

[36] Krunić J, Stojanović N, Đukić L, Roganović J, Popović B, Simić I, Stojić D (2019). Clinical antibacterial effectiveness and biocompatibility of gaseous ozone after incomplete caries removal. Clin. Oral Investig..

[37] Baysan A, Lynch E (2004). Effect of ozone on the oral microbiota and clinical severity of primary root caries. Am. J. Dent..

[38] Baysan A, Whiley RA, Lynch E (2000). Antimicrobial effect of a novel ozone-generating device on micro-organisms associated with primary root carious lesions in vitro. Caries Res..

[39] Al-Omiri MK, Alqahtani NM, Alahmari NM, Hassan RA, Al Nazeh AA, Lynch E (2021). Treatment of symptomatic, deep, almost cariously exposed lesions using ozone. Sci. Rep..

[40] Al-Omiri MK, Al Nazeh AA, Kielbassa AM, Lynch E (2018). Randomized controlled clinical trial on bleaching sensitivity and whitening efficacy of hydrogen peroxide versus combinations of hydrogen peroxide and ozone. Sci. Rep..

[41] Slee A, O'Connor J (1983). In vitro antiplaque activity of octenidine dihydrochloride (WIN 41464–2) against preformed plaques of selected oral plaque-forming microorganisms. Antimicrob. Agents Chemother..

[42] Zhang, Z., Mai, Y. & Yang, M. WebPower: Basic and advanced statistical power analysis. 0.5.2, R-Packages. https://CRAN.R-project.org/package=WebPower (2018).

[43] Gorelick L, Geiger A, Gwinnett A (1982). Incidence of white spot formation after bonding and banding. Am. J. Orthod..

[44] Kassambara, A. Ggpubr: ‘ggplot2’ Based Publication Ready Plots. 0.4.0, 2020. R-Packages. https://CRAN.R-project.org/package=ggpubr

[45] Practical Statistics in R for Comparing Groups: Numerical Variables. Independently published (2019).

[46] Rstatix: Pipe-Friendly Framework for Basic Statistical Tests. 0.7.0, 2021. R-Packages. https://CRAN.R-project.org/package=rstatix

[47] R: The R Project for Statistical Computing. https://www.r-project.org/. Accessed 17 Apr 2021.

[48] RStudio | Open Source and Professional Software for Data Science Teams—RStudio. https://www.rstudio.com/. Accessed 17 Apr 2021.

[49] Czaplińska J. Stan uzębienia, przyzębia i higieny jamy ustnej u dzieci i młodzieży leczonych aparatami stałymi. Rozprawa doktorska, Uniwersytet Medyczny im. K. Marcinkowskiego w Poznaniu (2012).

[50] Lindel I, Elter C, Heuer W, Heidenblut T, Stiesch M, Schwestka-Polly R, Demling A (2011). Comparative analysis of long-term biofilm formation on metal and ceramic brackets. Angle Orthod..

[51] Vicente A, Ortiz RA, González PB, García LJ, Bravo-González L (2017). Efficacy of fluoride varnishes for preventing enamel demineralization after interproximal enamel reduction Qualitative and quantitative evaluation. PLoS One.

[52] Śmiech-Słomkowska G, Jabłońska-Zarobek J (2003). Higiena jamy ustnej pacjentów leczonych stałymi aparatami ortodontycznymi. Mag. Stomat..

[53] Abbate G, Caria M, Montanari P, Mannu C, Orru G, Caprioglio A, Levrini L (2015). Periodontal health in teenagers treated removable aligners and fixed orthodontic appliances. J. Orofac. Orthop..

[54] Mizrahi E (1982). Enamel demineralisation following orthodontic treatment. Am. J. Ortod. Dentofac. Orthop..

[55] Mitchell L (1992). Decalcification during orthodontic treatment with fixe appliances—An overview. Br. J. Ortod..

[56] Øgaard B (1989). Prevalence of white spot lesions in 19-year-olds: A study on untreated and orthodontically treated persons 5 years after treatment. Am. J. Orthod. Dentofac. Orthop..

[57] Øgaard B, Bishara S, Duschner H, Graber T, Eliades T, Athanasiou A (2004). Enamel effects during bonding-debonding and treatment with fixed appliances. Risk Management in Orthodontics. Experts’ Guide to Malpractice.

[58] Singh S, Singh SP, Goyal A, Utreja AK, Jena AK (2016). Effects of various remineralizing agents on the outcome of post-orthodontic white spot lesions (WSLs): A clinical trial. Prog. Orthod..

[59] O’Reilly M, Featherstone J (1987). Demineralization and remineralization around orthodontic appliances: An in vivo study. Am. J. Orthod. Dentofac. Orthop..

[60] Øgaard B, Rezk-Lega F, Ruben J, Arends J (1992). Cariostatic effect and fluoride release from a visible light-curing adhesive for bonding of orthodontic brackets. Am. J. Orthod. Dentofac. Orthop..

[61] Arends J, Nelson D, Dijkman A, Jongebloed W, Gugenheim B (1984). Effect of various fluorides on enamel structure and chemistry. Cariology Today. International Congress.

[62] Shen P, Bagheri R, Walker G, Yuan Y, Stanton D, Reynolds C, Reynolds E (2016). Effect of calcium phosphate addition to fluoride containing dental varnishes on enamel demineralization. Aust. Dent. J..

[63] Tasios T, Papageorgiou SN, Papadopoulos MA, Tsapas A, Haidich AB (2019). Prevention of orthodontic enamel demineralization: A systematic review with meta-analyses. Orthod. Craniofac. Res..

[64] Richter A, Arruda A, Peters M, Sohn W (2011). Incidence of caries lesions among patients treat with comprehensive orthodontics. Am. J. Orthod. Dentofac. Orthop..

[65] Lovrov S, Hertrich K, Hirschfelder U (2007). Enamel demineralization during fixed orthodontic treatment—Incidence and correlation to various oral-hygiene parameters. J. Orofac. Orthop..

[66] Ahmed I, Saif-ul-Haque, Nazir R. Carious lesions in patients undergoing orthodontic treatment. *J. Pak. Med. Assoc*. **61**, 1176–1179 (2011). 22355961

[67] Enaia M, Bock N, Ruf S (2011). White-spot lesions during multibracket appliance treatment: A challenge for clinical excellence. Am. J. Orthod. Dentofac. Orthop..

[68] Jabłońska-Zarobek J, Śmiech-Słomkowska G (2005). Ryzyko próchnicy podczas leczenia ortodontycznego aparatem stałym. Czas. Stomatol..

[69] Staudt C, Lusi A, Jacquet J, Kiliardis S (2004). White spot lesionsaround brackets: In vitro detection by laser fluorescence. Eur. J. Oral Sci..

[70] Willmot D (2004). White lesions after orthodontic treatment: Does low fluoride make a difference?. J. Orthod..

[71] Arhun N, Arman A, Cehreli S, Arkan S, Karabulut E, Gulsahi K (2006). Microleakage beneath ceramic and metal bracketsbonded with a conventional and an antibacterial adhesive system. Angle Orthod..

[72] Benson P, Shah A, Millett D, Dyer F, Parkin N, Vine R (2005). Fluorides, orthodontics and demineralization: A systematic review. J. Orthod..

[73] Benson P, Shah A, Millet D, Dyer F, Parkin N, Vine R (2004). Some evidence that fluoride during orthodontic treatment reduces occurrence and severity of white spot lesions. Evid. Based Dent..

[74] Kronenberg O, Lussi A, Ruf S (2009). Preventive effect of ozone on the development of white spot lesions during multibracket appliance therapy. Angle Orthod..

[75] Huth KC, Paschos E, Brand K, Hickel R (2005). Effect of ozone on non-cavitated fissure carious lesions in permanent molars. A controlled prospective clinical study. Am. J. Dent..

[76] Al-Shamsi, A. Assessment of enamel changes during orthodontic treatment with and without ozone. PhD Thesis (School of Medicine and Dentistry Quue’s University of Belfast, 2007).

[77] Grocholewicz K, Matkowska-Cichocka G, Makowiecki P, Droździk A, Ey-Chmielewska H, Dziewulska A, Tomasik M, Trybek G, Janiszewska-Olszowska J (2020). Effect of nano-hydroxyapatite and ozone on approximal initial caries: A randomized clinical trial. Sci. Rep..

[78] Dogan A, Cetin E, Hussein E, Adiloglu A (2009). Microbiological evaluation of octenidine dihydrochloride mouth rinse after 5 days’ use in orthodontic patients. Angle Orthod..

[79] Askar H, Krois J, Rorher C, Mertens S, Elhennawy K, Ottolenghi L, Mazur M, Paris S, Schwendicke F (2021). Detecting white spot lesions on dental photography using deep learning: A pilot study. J. Dent..

